# Mimicking *in-vivo* exposures to drug combinations *in-vitro*: anti-tuberculosis drugs in lung lesions and the hollow fiber model of infection

**DOI:** 10.1038/s41598-019-49556-5

**Published:** 2019-09-13

**Authors:** Frank Kloprogge, Robert Hammond, Karin Kipper, Stephen H. Gillespie, Oscar Della Pasqua

**Affiliations:** 1 Institute for Global Health, University College London, London, United Kingdom; 2 School of Medicine, University of St Andrews, St Andrews, United Kingdom; 3 Analytical Services International Ltd, London, United Kingdom; 4 Clinical Pharmacology & Therapeutics Group, School of Pharmacy, University College London, London, United Kingdom

**Keywords:** Drug development, Translational research

## Abstract

Here, we evaluate protocol requirements to mimic therapeutically relevant drug concentrations at the site of infection (i.e. lung lesion) in an *in-vitro* hollow fibre model of infection using pulmonary tuberculosis as a paradigm. Steady-state pharmacokinetic profiles in plasma, lung tissue and lung lesion homogenate were simulated for isoniazid, rifampicin and pyrazinamide and moxifloxacin. An R-shiny User Interface was developed to support conversion of *in-vivo* pharmacokinetic C_MAX_, T_MAX_ and T_1/2_ estimates into pump settings. A monotherapy protocol mimicking isoniazid in lung lesion homogenate (isoniazid C_MAX_ = 1,200 ng/ml, T_MAX_ = 2.2 hr and T_1/2_ = 4.7 hr), and two combination therapy protocols including drugs with similar (isoniazid and rifampicin (C_MAX_ = 400 ng/ml)) and different half-lives (isoniazid and pyrazinamide (C_MAX_ = 28,900 ng/ml and T_1/2_ = 8.0 hr)) were implemented in a hollow-fiber system. Drug levels in the perfusate were analysed using ultra-high-performance liquid chromatographic-tandem mass spectrometric detection. Steady state pharmacokinetic profiles measured in the hollow fiber model were similar to the predicted *in-vivo* steady-state lung lesion homogenate pharmacokinetic profiles. The presented approach offers the possibility to use pharmacological data to study the effect of target tissue exposure for drug combinations. Integration with pharmacokinetics modelling principles through a web interface will provide access to a wider community interested in the evaluation of efficacy of anti-tubercular drugs.

## Introduction

The predictive value of nonclinical research for antimicrobial drug combinations, such as for tuberculosis, depends on how realistic experimental protocols reflect *in-vivo* conditions (e.g. nutrition, oxygen, drug exposure). This implies that experimental conditions must be tailored to the *in-vivo* disease conditions to ensure accurate translation of the findings. Whilst the use of the hollow fiber systems is not new, detailed research protocols are not readily available and it remains unclear whether such factors have been considered when results are reported in the literature. Of note are the challenges associated with regimens including drugs with different pharmacokinetic characteristics (e.g. different distribution, peak concentrations and different elimination half-lives) which need to be mimicked simultaneously within the experimental infection protocol using a single in one hollow fiber system. This oversight can have major implications for the dose rationale as well as the identification of suitable companion or partner compounds for combination therapy^1,2^.

This is relevant as combination therapy is common practice in the treatment of many infectious diseases, and essential in tuberculosis^3^. Yet, antibiotic combination regimens and doses have been based on empirical evidence of efficacy and safety. Attempts have been made to evaluate pharmacokinetics (drug concentration vs. time profiles) and pharmacodynamics (e.g. effect on mycobacterial load) *in-vivo* at the site of infection. However, experimental limitations make it difficult to fully characterise the concentration-effect relationship of each drug as well as their interaction when used as combination therapy. Serial pharmacokinetic and pharmacodynamic data may be difficult to obtain in a clinical setting and pulmonary tuberculosis is a good example of just such a challenging disease entitity. Data at lung lesions/granuloma level can usually only be obtained during a lung resection and not routinely due to the invasive nature of the procedure^4–6^.

Serial sampling for pharmacokinetics and pharmacodynamics could be achieved using the *in-vitro* hollow fiber model of infection^7^. Such protocols may enable the evaluation of a wide range of drug exposures^8^, mimicking drug concentrations likely to occur at the site of infection. This would be useful for novel antimicrobial compounds and combinations in pre-clinical stages and as a method to explore the efficacy of existing antimicrobial compounds being evaluated at different doses or in different combinations^9^.

The use of hollow fiber has been qualified by the EMA and the FDA as a technique that has value in the pre-clinical setting as complementary tool to existing anti-tuberculosis research methods^7,10^. As such, the method can be used to evaluate a variety of compounds in monotherapy or in differing combinations, allowing for a more comprehensive characterisation of bacterial killing effects based on factorial designs which remain rather complex, if not impossible, to conduct *in-vivo*^11^.

This investigation reports the protocol requirements to mimic therapeutically relevant drug concentrations at the site of infection (i.e. lung lesions). We have selected standard of care compounds (isoniazid, rifampicin, pyrazinamide and moxifloxacin) due to their clinical relevance and availability of pharmacokinetic and biopsy data^5^. We make the necessary settings and procedures available through a web-based application to the wider community.

## Materials and Methods

### *In-vivo* pharmacokinetic profiles

Steady-state total concentrations of isoniazid, rifampicin, pyrazinamide, and moxifloxacin were simulated using compartmental population pharmacokinetic models (Supplementary Material), which mimicked exposure profiles in plasma, lung tissue homogenate and lesion homogenate after standard oral doses (i.e. daily 600 mg rifampicin [450 mg for patients less than 50 kg in body weight], 300 mg isoniazid, 1500 mg pyrazinamide, and 400 mg moxifloxacin). A virtual population of 2,000 patients was simulated according to reported body weight distributions^12^. This sample size allowed us to evaluate the pharmacokinetic characteristics of the study drugs taking into account the covariate effects (i.e., body weight). For rifampicin, pyrazinamide and moxifloxacin 20 replicates of a virtual South Korean patient population of 100 patients were deemed sufficient to capture the overall variability in drug levels. For isoniazid, 20 replicates of a virtual population of 250 patients were used to ensure allocation of approximately 10% slow metabolisers across each body weight group^13^. Rifampicin auto-induction was accounted for by multiplying clearance estimates from a single dose model^14^ by a factor of 1.85. Geometric mean and 95% prediction intervals from the simulations were derived and visualised. Drug distribution from the systemic circulation to the site of infection was evaluated by comparing the ratio of lung/plasma exposure (R_tissue/plasma_ and R_lesion/plasma_) for isoniazid, rifampicin and pyrazinamide or rate-constants k_tissue-plasma_, k_plasma-tissue_, k_lesion-plasma_ and k_plasma-lesion_ for moxifloxacin (see appendix for further details).

### R Shiny web application

An R-shiny web application, was developed (https://pkpdia.shinyapps.io/hfs_app/) to facilitate the conversion of *in-vivo* pharmacokinetic profiles into *in-vitro* pump settings in the hollow fiber system. The application supports the generation of pharmacokinetic profiles after monotherapy and combination therapy by converting key pharmacokinetic parameters C_MAX_, T_MAX_ and T_1/2_ into pump settings using a published method running at the back end^15^.

**Figure 1:**
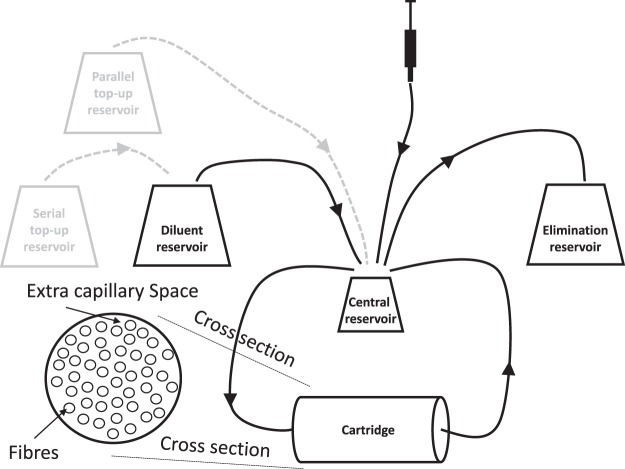
Schematic representation of a hollow fiber protocol mimicking exposure to drug combinations at the site of infection. Grey boxes and dashed lines represent top-up reservoirs and tubing for experiment for the implementation of an experiment with two drugs showing different elimination half-lifes.

The pump rate for the diluent, elimination, and titration reservoir (including scenarios in which two drugs with different half-lives are used) was calculated as: ${V}_{system}\times \frac{LN(2)}{{t}_{1/2}}$, where t_1/2_ represents the elimination half-life of the study drug and V_system_ represents the hollow fiber system volume (i.e. central reservoir, cartridge and tubes). Drug amounts required to mimic a certain C_MAX_ were calculated by: C_MAX_ × V_system_ where C_MAX_ represents the maximum concentration. Oral absorption was based on syringe pump rates calculated by: $\frac{{C}_{MAX}\times {V}_{system}}{{t}_{MAX}}$, where t_MAX_ is the time associated with C_MAX_. Simultaneous simulation of a short and a long half-life drug in the *in-vitro* hollow fiber was achieved by topping up the amount in the long half-life drug in the diluent reservoir (i.e. serial setting) or by an additional reservoir (i.e. parallel setting). Subsequently, the pump-rate settings from the dilution reservoir were adjusted accordingly in order to ensure that the R_dilution reservoir_ + R_titration reservoir_ was identical to R_elimination reservoir_ (Fig. 1).

### *In-vitro* pharmacokinetic profiles

Pump settings for three antibiotics, which are commonly used as part of the standard tuberculosis treatment combination, were calculated by a web-application (https://pkpdia.shinyapps.io/hfs_app/) assuming a total system volume of 75 ml. Pharmacokinetic profile characteristics in lung lesions (i.e. C_MAX_, T_MAX_ and T_1/2_) for the three antibiotic drugs, based on standard dosing regimens, were selected and accounted for plasma protein binding (i.e. 42% for isoniazid, 83% for rifampicin and 10% for pyrazinamide)^16^. Three experimental conditions were tested. One monotherapy experiment with isoniazid at C_MAX_ = 1,200 ng/ml, T_MAX_ = 2.2 hour and T_1/2_ = 4.7 hour was conducted. A combination therapy experiment with similar half-life drugs was conducted using isoniazid at C_MAX_ = 1,200 ng/ml and rifampicin at C_MAX_ = 400 ng/ml with T_MAX_ = 2.2 hour and T_1/2_ = 4.7 hour. Lastly, a combination therapy experiment with two drugs showing different half-lives was conducted using isoniazid at C_MAX_ = 1,200 ng/ml, T_MAX_ = 2.2 hour and T_1/2_ = 4.7 hour and pyrazinamide at C_MAX_ = 28,900 ng/ml, T_MAX_ = 2.2 hour and T_1/2_ = 8.7 hour. All experiments were performed under uninfected conditions at 37 °C. Rifampicin and pyrazinamide T_MAX_ were set to isoniazid T_MAX_ in order to avoid an overly complex experimental setup even though clinical rifampicin and pyrazinamide T_MAX_ are different. Rifampicin T_1/2_ was harmonised to isoniazid to serve an example of an experiment of drugs with similar half-lives and T_MAX_ and T_1/2_ values were derived based on the pharmacokinetic model for isoniazid. Subsequent experimental dilution mistakes in the solution to be infused into the central reservoir (i.e. 10, 100 and 2-fold for isoniazid, rifampicin and pyrazinamide), only affecting C_MAX_, were accounted for as the pump settings were correctly set and therefore T_MAX_ and T_1/2_ remained unaffected. The mean Prediction Error (MPE) was calculated ($MPE=\frac{{\sum }_{i=1}^{n}\,D{V}_{i}-IPRE{D}_{i}}{n}$) with the dependent variable (DV) and model predictions (IPRED) as numerical measure of bias of the pharmacokinetic profiles generated in the hollow fiber model of infection. The MPE was used together with visual inspection to assess how well the *in-vitro* pharmacokinetic profiles approximated the dashed line that represented those generated by the user. The simulated *in-vivo* lung lesion homogenate profiles for a typical patient with 95%-prediction intervals, taking into account plasma protein binding, were overlaid to ensure that the simulated *in-vitro* profiles fell within the prediction intervals. For isoniazid a typical patient with a clearance at the lower end of the distribution, at 13.1 l/hr, was used to mimic a slow metaboliser^17^.

The integrated hollow fiber experimental protocol comprised a FiberCell Systems Duet pump with a cellulosic cartridge (medium sized – C3008). Drugs were dissolved in 1 ml broth and infused into the central reservoir to mimic an oral absorption profile using a NE-4000 Programmable 2 Channel Syringe Pump. Flow rates, to generate *in-vivo* mimicking drug elimination profiles, were controlled using a Masterflex C/L Dual-Channel Pump and Masterflex SC0033-LT Tygon E-Lab ext tubing. Samples (2 ml) were taken just before drugs were added to the experiment and 2.2, 5, 8 and 24 hours after, both from the central reservoir and from the extra-capillary space of the cartridge (Fig. 1).

### Ultra-high-performance liquid chromatographic-tandem mass spectrometric detection of analytes

Bioanalysis of drug concentrations was performed by chromatographic separation and mass spectrometric detection of three analytes using Acquity ultra-high-performance liquid chromatography (UPLC) system equipped with Waters TQ Detector (Waters, Milford, USA). The UPLC system consists of a binary solvent manager, a sample manager and a column thermostat. ESI-MS detection was carried out in positive ion detection mode. The assay was linear (r^2^ > 0.993) for isoniazid and rifampicin over the concentration range from 10 ng/mL to 10,000 ng/mL and from 10 ng/mL to 50,000 ng/mL for pyrazinamide. An appropriate dilution scheme was used for sample analysis to match the calibration concentrations. Detailed description on the sample preparation and a LC-MS/MS assay is presented in the Appendix section; Quantification of analytes *in-vitro* pharmacokinetic profiles.

## Results

### *In-vivo* pharmacokinetic profiles

**Figure 2:**
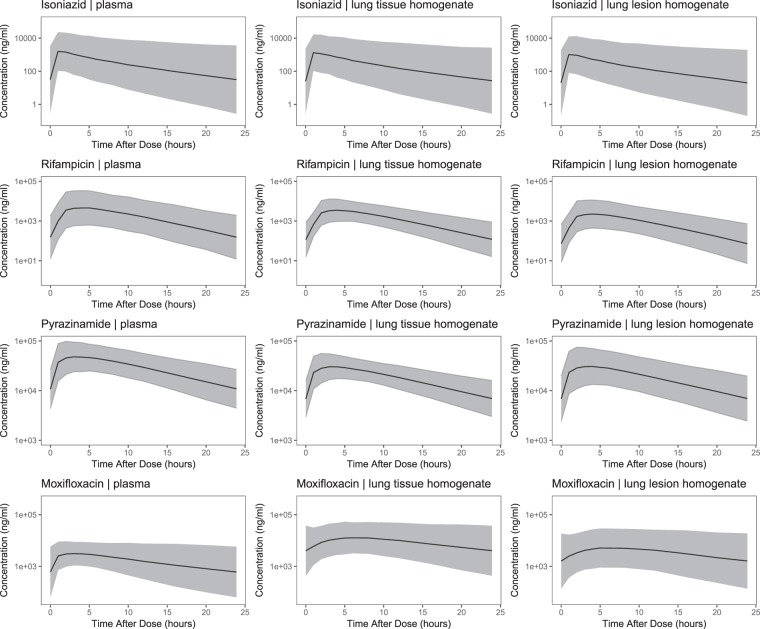
Simulated steady-state total isoniazid, rifampicin, pyrazinamide and moxifloxacin in plasma, lung tissue and lesion homogenate. Solid black lines represent the geometric mean of 2,000 simulated patients. Shaded areas represent the 95% prediction intervals.

Simulations using the population pharmacokinetic models for isoniazid, rifampicin and pyrazinamide displayed rapid distribution from the plasma into healthy lung tissue homogenate and lung lesion homogenate. It also suggested that lower exposure in the lung tended to remain within variability of the plasma data (Fig. 2). Total drug concentrations in the lung tissue and lung lesion homogenate are lower for isoniazid (9.3% [RSE: 23.5%] and 29.8% [RSE: 21.7%]), rifampicin (24.8% [RSE: 15%] and 52.2% [RSE: 13%]) and pyrazinamide (37.2% [RSE: 7%] and 36.8% [RSE: 8%]) as compared to plasma concentrations (Appendix Table 1). Unlike isoniazid, rifampicin and pyrazinamide, simulations of the profiles of moxifloxacin revealed a nearly doubled elimination half-life in lung tissue and lung lesion homogenate as compared to plasma. This resulted from a substantially faster distribution and diffusion of total moxifloxacin concentrations from plasma to lung tissue (k_24_ = 1.584 h^−1^ [37.3% RSE]) or lung lesion (k_25_ = 0.633 h^−1^ [79.8% RSE]) as compared to the re-distribution and clearance from lung tissue (k_42_ = 0.314 h^−1^ [50.1% RSE]) or lung lesion (k_52_ = 0.318 h^−1^ [73.3% RSE]) back to the plasma (Appendix Table 1). The geometric mean ± 1.96 SD of the simulated steady state profiles displayed in Fig. 2 show the between patient variability, which was determined primarily by the plasma data as time series data for lung tissue or lesion homogenate were not available (Appendix Fig. 1). Detailed information about the population pharmacokinetic model development and performance are summarised in the appendix (Appendix Table 1 and Fig. 1).

### Facilitating modelling of pharmacokinetic profiles *in-vitro*

**Figure 3:**
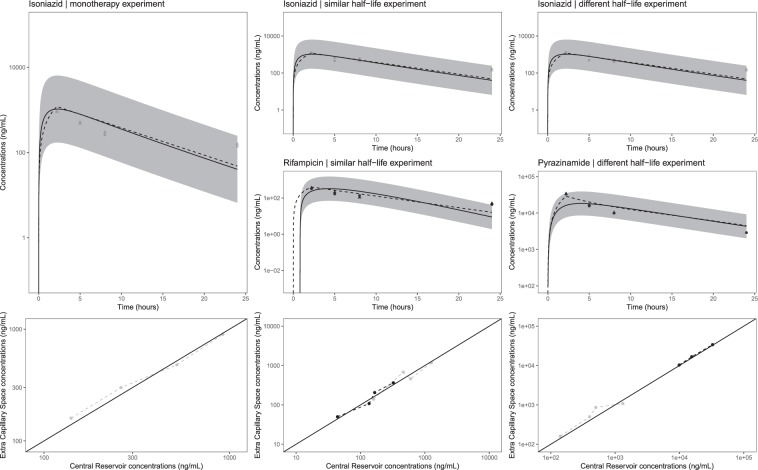
Overview of *in-vitro* pharmacokinetic profiles obtained in the hollow fiber system for isoniazid monotherapy, isoniazid + rifampicin combination therapy and isoniazid + pyrazinamide combination therapy. Data relflect drug levels after correction for plasma protein binding. Circles and triangles in the concentration vs. time profile plots (top panels) represent single samples in the central reservoir and extra capillary space concentrations, respectively. Dashed lines represent the predicted *in-vitro* pharmacokinetic profiles obtained by the user interface, which is required for the selection of the pump settings. Shaded areas and solid lines in the concentration vs. time profile plots (top panels) represent the geometric mean and 95%-predicition intervals of a typical profile in humans. The bottom panels show the drug concentrations in the central reservoir and extra capillary space in the hollow fiber system.

The web application was used to convert secondary pharmacokinetic parameter estimates C_MAX_, T_MAX_ and T_1/2_ into pump settings to mimic lung lesion homogenate exposure taking into account protein binding. We have evaluated three antibiotics from the standard tuberculosis treatment combination, including isoniazid, isoniazid plus rifampicin (i.e. same half-lives) and isoniazid plus pyrazinamide (i.e. different half-lives) (Table 1). Prior to T_MAX_ was reached (in all experiments) drugs were infused using a zero-order process into the central reservoir and after T_MAX_ was reached flow rates between the diluent and central reservoir, and central and elimination reservoirs were set at an identical rate. The pharmacokinetic profiles of pyrazinamide (half-live 8.02 hour) and isoniazid (half-live 4.7 hour) were considerably different and this could be adjusted for through a serial top up setting (Fig. 1). The flow rate between the serial top-up reservoir and diluent reservoir was equal to the flow rate between the diluent reservoir and the central reservoir (Table 1). For the example with drugs showing similar half-lives, top-up reservoirs (neither serial nor parallel) were needed (Table 1). The *in-vitro* pharmacokinetic profiles, accounted for protein binding, tended to show over prediction for isoniazid and rifampicin at the last sampling time point. However, the general trend for the three experiments was acceptable and remained within the prediction intervals for lung lesion homogenate (Fig. 3). The isoniazid MPE was −178, −20.6, and −18.1 ng/ml for the monotherapy, rifampicin combination therapy and pyrazinamide combination therapy experiment, respectively. The rifampicin and pyrazinamide MPE was −37.9 and −1397 ng/ml, respectively. All three drugs displayed adequate penetration of the fibres yielding similar exposure in the central reservoir and extra-capillary space (Fig. 3).

**Table 1:** Overview of secondary parameter conversions into pump setting for selected hollow fiber experiments.

Parameter	Isoniazid	Isoniazid -rifampicin	Isoniazid -pyrazinamide
C_MAX isoniazid_ (ng/ml)*	1,200	1,200	1,200
C_MAX companion drug_ (ng/ml)*	—	400	28,900
T_MAX_ (hour)	2.15	2.15	2.15
T_1/2 isoniazid_ (hour)	4.7	4.7	4.7
T_1/2 companion drug_ (hour)	—	4.7	8.02
Flow rate dilution/serial top-up/elimination reservoir (ml/min)	0.18	0.18	0.18
Flow rate dilution/serial top-up/elimination reservoir start time (min)	129	129	129
Amount _isoniazid_ (ug)	90	90	90
Amount _companion drug_ (ug)	—	30	2,168
Syringe infusion rate _isoniazid_ (ug/min)	0.70	0.70	0.70
Syringe infusion rate _companion drug_ (ug/min)	—	0.23	16.8
Amount _companion drug serial top-up reservoir_ (ug)	—	—	4,247
Dilution/serial top-up reservoir (ml)	265	265	265

*Accounted for protein binding.

## Discussion

In this paper we report an *in-vitro* hollow fiber model of infection integrated with a pharmacokinetic model as a tool for the evaluation of target tissue exposure. Given the complexity of incorporating organ and plasma disposition data into a pharmacokinetic model and implications for infusion rates and titration steps, a user interface was also developed. This interface provides a simple way for investigators to use the hollow fiber in their own investigations.

The *In-vitro* pharmacokinetic profiles for lung lesion homogenate generated in the hollow fiber system fell within the intervals predicted by the population pharmacokinetic models taking into account plasma protein binding *in-vivo*. The *in-vitro* pharmacokinetic profiles generally followed the simulated *in-vivo* steady-state lung lesion homogenate profiles. However, a trend of higher *in-vitro* generated concentrations was observed for the later measurements of isoniazid and rifampicin, as compared to the web-application based simulations (Fig. 3). The discrepancies could probably be attributed to the variability in the peristaltic pump and sampling handling. Reproducibility of pharmacokinetic profiles (i.e. between experiment variability) was acceptable, as isoniazid levels in all three experiment were comparable. One way to account for between lab/setup variability, in addition to ensuring accurate calibration of the pumps, is to analyse the *in-vitro* pharmacokinetic profiles in conjunction to bacterial load level data from the same experimental protocol. Simultaneous modelling of pharmacokinetic and pharmacodynamic data may be especially beneficial when experimental variability is a concern.

In order to avoid over complicating the experimental setup with series of syringe drivers at different infusion rates we have simulated drug levels that correspond to the terminal elimination phase, even though isoniazid displays bi-phasic disposition pharmacokinetics *in-vivo*. This resulted in a monophasic pharmacokinetic profile *in-vitro*. Future experiments may consider more realistic scenarios, including repeated rifampicin dosing, which displays auto-induction. Whilst more complex, these scenarios imply further modification of the pump setting during the course of the experiment. Furthermore, our method allowed to standardise T_MAX_ for companion drugs to the treatment backbone, i.e. in this example rifampicin and pyrazinamide were coupled to the same value of T_MAX_ of isoniazid to simplify the experimental setup. Although *in-vivo* oral absorption is a first order process, for simplification we have adopted an experimental setup for *in-vitro* oral absorption with a zero-order infusion.

Mimicking representative environmental parameters remains key for the interpretation of data arising from the hollow-fiber model for mycobacterial infections. In the current study, we did not include the contribution of the inoculum as factor to be considered, but the approach proposed here can be used with different drugs and any other aspect of an experimental infection protocol. Moreover, tuberculosis for example has three metabolic states, the log-phase, acid-phase and non-replicative persister phenotype-phase which all tend to reside in different environments. An acid-phase organism often stays within the macrophage whereas the non-replicative persister phenotype-phase tends to reside in the caseum, which renders the environmental pH important. This can be modelled in the hollow-fibre model of infection by modifying the broth, oxygen levels in the incubator, flow rates and adding macrophages. However, this also has implications for drug levels to be simulated. When one uses homogenate levels it is not possible to distinguish and quantify intra- and extra-cellular levels. It is also difficult to assess whether drug levels were similar in the caseum or at the edges of the lesion.

Serial *in-vivo* exposure vs. time data remains key to inform dose selection for experimental protocols using the hollow fiber system. Drug level data is, however, often quantified as total drug concentrations (i.e. protein unbound plus protein-bound) even though it remains questionable whether total drug levels are the most suitable metrics for further characterisation of pharmacokinetic-pharmacodynamic relationships. Depending on the mechanism of action, possibly free drug concentrations at the site of infection might be more relevant for the antibacterial activity, e.g., rifampicin that prevents *Mycobacterium tuberculosis* to grow by inhibiting DNA-dependent RNA synthesis^18^. However, unlike in plasma, little^6^ or no *in-vivo* information is available on drug protein binding or non-specific tissue binding at the site of infection in patients (i.e. lung lesions in the case of pulmonary tuberculosis). Total homogenate concentrations could also be misleading in that it does not represent a demarcated physiological compartment, hence in homogenates all cell boundaries have been destroyed, which results in mixed intra- and extra-cellular concentrations^19^. Often, as only total drug concentrations are available, assumptions are required to conduct a hollow-fiber experiment to ensure other factors such as protein- and/or tissue-drug binding are taken into consideration. First, one could assume that due to poor vascularisation of the lung lesion the equilibrium of free drug between plasma and lung lesions is impaired. A best guess for this scenario is complex, if not impossible, as free drug levels at lung lesion homogenate would be needed. To evaluate this scenario in the current study, total lesion homogenate concentrations were used, after correction for plasma protein binding. Another scenario would assume that free drug can cross the blood vessel and cell membranes yielding free drug concentrations in plasma and lung lesion to be in equilibrium. However, this assumption might not be representative of necrotic foci of the lesion. Alternatively, physiochemical characteristics could be taken into account (e,g, sum of the hydrophobicity and number of aromatic rings), to describe drug distribution and binding to caseum^20^. In fact, very similar fractions of unbound drug in plasma and caseum for isoniazid, rifampicin and pyrazinamide, were found in *in-vitro* experiments mimicking caseum and lung lesion and homogenate from rabbits. These findings seem in agreement with our results. Clearly, the scenario in this study where total lesion homogenate concentrations is used might lack translational value, but one needs to acknowledge that each of the alternative proposed methods also have their drawbacks. Only *ex-vivo* microdialysis data may provide information on free drug levels at the site of infection, although this type of data is rarely available^6^.

It is important to highlight that assumptions will have to be made on the drug concentration vs. time profiles when the hollow fiber system is used to evaluate novel antibiotic drug combinations for which human pharmacokinetic data is unavailable. Lung lesion drug levels from animal experiments can and should be used in these circumstances. Alternatively, when only plasma level data are available in healthy subjects, predictions of lesion and tissue exposure may be considered using physiochemical properties and physiological-based pharmacokinetic models^20^.

In summary, we have shown how pharmacokinetic profiles of drugs combinations in humans can be reproduced in the hollow fiber system, mimicking drug levels at the target tissue. We have created an R-shiny interface for other users. Our approach offers an opportunity for drug combinations to be evaluated more thoroughly in the early phases of drug development eliminating some of the uncertainties that limit the evaluation of relevant doses during clinical trials.

## Supplementary information

Supplementary material for: Mimicking in-vivo exposures to drug combinations in-vitro: anti-tuberculosis drugs in lung lesions and the hollow fiber model of infection.

## Data Availability

Data generated and analysed during the current study are available from the corresponding author on reasonable request.
