# Supplementary material for: Mimicking in-vivo exposures to drug combinations in-vitro: anti-tuberculosis drugs in lung lesions and the hollow fiber model of infection
Source: Sci Rep. 2019 Sep 13;9:13228. doi: 10.1038/s41598-019-49556-5 (PMC6744479; doi:10.1038/s41598-019-49556-5)
Supplement: Supplementary file 1 — Supplementary material for: Mimicking in-vivo exposures to drug combinations in-vitro: anti-tuberculosis drugs in lung lesions and the hollow fiber model of infection. [file 41598_2019_49556_MOESM1_ESM.docx]

Supplementary material for: Mimicking *in-vivo* exposures to drug combinations *in-vitro*: anti-tuberculosis drugs in lung lesions and the hollow fiber model of infection.

Frank Kloprogge^1^, Robert Hammond^2^, Karin Kipper^3^, Stephen H. Gillespie^2^ and Oscar Della Pasqua^4^

^1^ Institute for Global Health, University College London, London, United Kingdom

^2^ School of Medicine, University of St Andrews, St Andrews, United Kingdom

^3^ Analytical Services International Ltd, London, United Kingdom

^4^ Clinical Pharmacology & Therapeutics Group, University College London, London, United Kingdom

# Corresponding author:

Frank Kloprogge

UCL Institute for Global Health

3^rd^ floor Institute of Child Health

30 Guilford Street

London

WC1N 1EH

Email: [f.kloprogge@ucl.ac.uk](mailto:f.kloprogge@ucl.ac.uk)

Contents

[Quantification of drug concentrations *in vitro* 2](#_Toc525146395)

[Chemicals and standards 2](#_Toc525146396)

[Sample preparation 2](#_Toc525146397)

[Ultra-high-performance liquid chromatographic-tandem mass spectrometric detection of analytes 2](#_Toc525146398)

[Development of population pharmacokinetic models 3](#_Toc525146399)

[Pharmacokinetic data preparation and formatting 3](#_Toc525146400)

[Population pharmacokinetic modelling methods 4](#_Toc525146401)

[Population pharmacokinetic modelling results 5](#_Toc525146402)

[Isoniazid 5](#_Toc525146403)

[Rifampicin 6](#_Toc525146404)

[Pyrazinamide 7](#_Toc525146405)

[Moxifloxacin 8](#_Toc525146406)

[Population pharmacokinetic modelling discussion 11](#_Toc525146407)

[References 12](#_Toc525146408)

# Quantification of drug concentrations *in vitro*

## Chemicals and standards

Isoniazid was obtained from LKT Laboratories Inc. (St. Paul, USA), whereas rifampicin was obtained from Fargon UK Ltd (Newcastle Upon Tyne, UK). Pyrazinamide and Middlebrook 7H9 broth base, NaHCO_3_, CH_2_Cl_2_, isopropanol and LC-MS grade methanol and formic acid were purchased from Sigma (St. Louis, USA).

Internal standards - [^13^C_2_,^15^N_2_]-pyrazinamide, isoniazide-D_4_, and rifampicin-D_8_ were obtained from Alsachim (Illkirch Graffenstaden, France). Water was purified (18.2 MΩ*cm at 25 °C and a TOC (total organic carbon) value below 3 ppb) in house using a Millipore Advantage A10 system from Millipore (Bedford, USA).

## Sample preparation

Matrix matched calibration and stable isotope labelled internal standards (IS) were used to quantify drug concentration. Analytes were extracted from 500 µL aliquots by liquid-liquid extraction. 300 µL of internal standard mixture in methanol (containing 5 µg/mL of [^13^C_2_,^15^N_2_]-pyrazinamide, 1 µg/mL of isoniazide-D_4_, and 1 µg/mL of rifampicin-D_8_) and 300 µL of NaHCO_3_ buffer with pH 8.5 were added to samples following 2.5 mL of CH_2_Cl_2_/isopropanol (50/50). Thereafter samples were mixed for 10 minutes and centrifuged at 3000 x g. Solvent layer was evaporated to dryness on a SpeedVac Concentrator at low temperature settings. Samples were reconstituted in 100 µL of 2 % MeOH and submitted to analysis by ultra-high-performance liquid chromatography – tandem mass spectrometry (UPLC-MS/MS) using Waters Acquity Ultra High Performance Liquid Chromatography (UPLC) system equipped with Waters TQ Detector (Waters, Milford, USA).

## Ultra-high-performance liquid chromatographic-tandem mass spectrometric detection of analytes

A novel methodology was developed for the chromatographic separation of isoniazid, rifampicin, and pyrazinamide as existing methods were suitable for plasma samples whereas in this study broth samples were analyses ^1,2^. Separation occurred using 0.1% formic acid in water (mobile phase A) and 0.1% formic acid in methanol (mobile phase B) with gradient elution and a reversed phase analytical column (50mm x 2.1mm; 1.7 µm Acquity UPLC BEH C18 (Waters, Milford, USA). Separation was obtained using the gradient program starting from 3% of mobile phase B for the first minute. Thereafter, mobile phase B contents was raised to 100% over one minute and kept at 100% for 0.8 minutes, then lowered again to 3% over 0.7 minutes and kept at 3% for 0.5 minutes. Eluent flow rate was 0.25 mL/min and injection volume 20 µL. Electrospray interface (ESI) was used for the mass-spectrometric detection in the positive multiple reaction monitoring (MRM) mode for detection of analytes. Triple quadrupole detector transitions *m/z* 124.02 [M+H]^+^ -> *m/z* 106.93; 51.96 (for pyrazinamide); *m/z* 137.98 [M+H]^+^ -> *m/z* 120.90; 78.82 (for isoniazid); *m/z* 823.53 [M+H]^+^ -> *m/z* 151.00; 791.35 (for rifampicin); *m/z* 127.91 [M+H]^+^ -> *m/z* 110.95 (for [^13^C_2_,^15^N_2_]-pyrazinamide, IS); *m/z* 142.06 [M+H]^+^ -> *m/z* 125,05 (for isoniazide-D_4_, IS); and *m/z* 831.62 [M+H]^+^ -> *m/z* 799.52 (for rifampicin-D_8_, IS) were used for quantification and qualification.

Optimised parameters for ESI and mass spectrometer were used with capillary voltage of 4 kV, cone voltage of 25 V, source temperature of 150°C, desolvation gas temperature of 250°C and flow rate of 500 L/h and cone gas flow rate of 30 L/h. For analyte quantification, calibration curves were created using linear regression with a weighting factor of 1/x^2^ Quality control (QC) samples at three concentration levels (50 ng/mL, 1000 ng/mL and 7000 ng/mL) were included in the beginning, middle and end of the batch. The UPLC-MS/MS method was validated for linearity, accuracy and precision. Within-day and between-day accuracies ranged from 92.0% – 110.8% for all analytes at all QC levels, within-day and between-day precision ranged from 4.6% to 9.9 % for all analytes at all QC levels.

# Development of population pharmacokinetic models

## Pharmacokinetic data preparation and formatting

Healthy lung tissue as well as lung lesion homogenate data were reported in ng/g tissue, whereas plasma concentrations were reported in ng/ml^3^. Healthy lung tissue and lung lesion homogenate data were therefore converted to ng/ml, under the assumption that 1 g of tissue corresponds to a volume of 1mL of tissue homogenate.

Patients were on a variety of antibiotic drug combinations in the background and consequently one or more of the study drugs (i.e. isoniazid, rifampicin, pyrazinamide and moxifloxacin) might have been administered as single dose or as part of the background therapy, which was accounted for in the population pharmacokinetic models ^3^. However, a few inconsistencies in the reported background treatment and dose schedules were identified. Patient number 9 (G110) was reported to have received moxifloxacin and pyrazinamide background therapy in the covariate summary although in the raw concentration-time data files this patient appeared to have received a single dose for either moxifloxacin and pyrazinamide. Similarly, patient 14 (G402) was reported to have received moxifloxacin background therapy in the raw concentration-time data files, but this did not reflect the data shown in the covariate summary. To overcome this contradiction, baseline concentrations for patient 9 (G110) were estimated but not coded as steady-state. In addition, no baseline concentrations were estimated for patient 14 (G402).

## Population pharmacokinetic modelling methods

A population pharmacokinetic analysis was conducted to gain further insight into drug levels in lung tissue and lesion, and thereby to support the generation of pharmacokinetic profiles *in-vitro* that mimic *in-vivo* drug disposition. The analysis consisted in the evaluation of publicly available individual patient level data for the standard of care drugs included in this manuscript (isoniazid, rifampicin, and pyrazinamide) and moxifloxacin^3^. The study comprised fifteen South Korean patients with multi drug resistant tuberculosis scheduled for partial lung removal. All patients received a single oral dose of 600mg rifampicin (450 mg for patients less than 50 kg body weight), 300 mg isoniazid, 1500 mg pyrazinamide, and 400 mg moxifloxacin. Dosing times were 2, 4, 8, 12 or 24h prior to scheduled surgery with 3 subjects randomized to each of the 5 target time points. Drug concentrations were measured in samples in plasma, healthy lung tissue and lung lesion homogenate. Due to the wide range of concentrations, drug levels in plasma, lung tissue and lesion homogenates were modelled in their natural logarithm using NONMEM v. 7.3.0 with a gfortran compiler on a windows 10 operating system. Minus twice the log likelihood of the data was used as objective function value (OFV) and approximations to the true likelihood were obtained using the First Order Conditional Estimation method with Interaction (FOCE-I). Pirana v. 2.9.0 (Pirana Software & Consulting BV), PsN v. 4.2.0 (http://psn.sourceforge.net/) and R v. 3.2.3 (https://www.r-project.org/) were used, respectively, for model building, data manipulation, graphical and statistical summaries.

Model building was performed as follows for all paradigm drugs:

First, a pharmacokinetic model empirically describing the plasma concentration data (step I) was developed and compared to pharmacokinetic models including prior parameter distributions (step II) and models where parameters were fixed to literature values (step III). If possible, published models based on a study protocol design covering a 0-24 h post-dose sampling window were used to inform prior parameter distributions and uncertainty on parameters was included as variance, derived from Relative Standard Errors on published parameter estimates (models in step II). Parameters were fixed to literature values if pharmacokinetic parameter estimates (e.g. absorption and/or distribution parameters) were not identifiable (models in step III).

The best performing and most parsimonious model structure was carried forward for the estimation of drug concentrations in lung tissue and lesion. Data from plasma, lung tissue and lesion homogenate was analysed using an empirical compartmental model structure^4^ for drugs showing no accumulation in lung tissue or lesion (i.e. isoniazid, rifampicin and pyrazinamide). By contrast, a more complex model structure^5^ was deemed necessary for moxifloxacin to account for accumulation in tissue and lesion. PK parameter estimation was based on simultaneous fitting of plasma, lung tissue and lesion data whenever feasible (e.g. pyrazinamide). Alternatively, a two-stage, sequential approach was used in which lung tissue and lung lesion data were modelled using individual predicted plasma concentrations as input.

Model performance was assessed using basic goodness of fit plots (i.e. observation vs. individual prediction, observations vs. population prediction, conditional weighted residuals vs. population prediction and conditional weighted residuals vs. time), population and individual prediction vs. time overlaid with observed concentrations over time and objective function value (i.e. -2 log-likelihood between hierarchical models with a 3.84 (p<0.05) drop as significant improvement between hierarchical models). In addition, visual predictive checks based on simulated plasma, lung tissue and lesion concentrations (n=2000) were implemented, by overlaying the 95%-prediction intervals of the 5^th^, 50^th^ and 95^th^ percentiles with the observed data (5^th^, 50^th^ and 95^th^ percentiles of the concentration vs. time profile). At last, the robustness of the parameter estimates of the final model was also assessed by bootstrapping (n=1000).

## Population pharmacokinetic modelling results

### Isoniazid

Total isoniazid concentration-time profiles were reasonably well described using a two-compartment model with first-order absorption and first-order elimination (Supplement Figure 1; Supplement Table 1). Distribution model parameter estimates were supported by informative priors from published literature and, due to the absence of acetylator-status information, clearance was parameterised as uninformative prior ^6^. Apart from elimination clearance, posterior estimates differed only marginally from the prior estimates. We have not considered this to be an indication of model misspecification. Most likely, it reflects the limited informative content of the sparse data. Total isoniazid concentration-time profiles in lung tissue homogenate and lung lesion homogenate were determined by Empirical Bayes Estimates (EBE) from the plasma model and an empirical effect site compartment for lung tissue and lesion homogenate with an additive residual error on log transformed data (Supplement Figure 1; Supplement Table 1).

|  |
| --- |
| Supplement Figure 1. Visual predictive check for isoniazid. The solid line represents the median, whereas the dashed lines represent the 5^th^ and 95^th^ percentiles of the observed (black dots) data. Dark and light grey shaded areas represent the 95% prediction intervals of the median, 5^th^ and 95^th^ percentiles of the predicted concentrations (N_simulations_=2000). |

### Rifampicin

Total rifampicin concentration-time profiles were reasonably well described using a one-compartment model with first-order absorption and first-order elimination (Supplement Figure 2; Supplement Table 1). Apparent volume of distribution and absorption rate constant as well as inter-individual variability on the apparent volume of distribution volume were fixed to published literature values^7^. However, model fitting with published parameter values as priors displayed worse performance^7,8^ (results now shown). Unlike the three other drugs, for which only one patient was poorly described, rifampicin profiles were note adequately described for another two individuals. Drug clearance in these subjects was too low, resulting in an overestimation of the ratio between plasma vs lung tissue homogenate or lung lesion homogenate. As a result, the PK model describing rifampicin plasma concentrations was refitted after exclusion of these data (patients 1, 8 and 12). EBE parameter estimates were subsequently used to describe the individual concentration-time profiles in lung tissue homogenate and lung lesion homogenate. Total rifampicin tissue concentrations (for lung tissue and lesion homogenate) was characterised by an empirical effect site compartment with an additive residual error on log transformed data (Supplement Figure 2; Supplement Table 1).

|  |
| --- |
| Supplement Figure 2. Visual predictive check for rifampicin. The solid line represents the median, whereas the dashed lines represent the 5^th^ and 95^th^ percentiles of the observed (black dots) data. Dark and light grey shaded areas represent the 95% prediction intervals of the median, 5^th^ and 95^th^ percentiles of the predicted concentrations (N_simulations_=2000). |

### Pyrazinamide

Total pyrazinamide concentration-time profiles were reasonably well described using a one-compartment disposition model with first-order absorption and elimination (Supplement Figure 3; Supplement Table 1). The model did not benefit from a more complex sequential zero-order followed by first-order absorption model with two populations, representing fast and slow absorbers, together with bodyweight and gender effect on clearance and volume of distribution, supported with priors (data not shown)^9^. Simultaneous estimation of plasma data with total lung tissue homogenate and total lung lesion homogenate data in an empirical effect site compartment with additive residual error terms on log transformed data for all matrices provided acceptable performance. There was no improvement in the fitting by sequential analysis of lung tissue and lung lesion homogenate concentrations using plasma EBE estimates.

|  |
| --- |
| Supplement Figure 3. Visual predictive check for pyrazinamide. The solid line represents the median, whereas the dashed lines represent the 5^th^ and 95^th^ percentiles of the observed (black dots) data. Dark and light grey shaded areas represent the 95% prediction intervals of the median, 5^th^ and 95^th^ percentiles of the predicted concentrations (N_simulations_=2000). |

### Moxifloxacin

Total moxifloxacin concentration-time profiles were reasonably well described using a two-compartment model with first-order absorption and first-order elimination (Supplement Figure 4; Supplement Table 1). Distribution model parameters were fixed to values in published literature^10^. A simplified one compartment model with first order absorption and elimination displayed worse performance (data not shown). Individual total concentration-time profiles in lung tissue homogenate and lung lesion homogenate and residual additive variability on log transformed data was estimated simultaneously using plasma EBE parameter estimates. As distribution of moxifloxacin from plasma to the lung tissue was substantially faster (k_24_=1.584 [37.3% RSE]) than from lung tissue back to plasma (k_42_=0.314 [50.1% RSE]), higher moxifloxacin concentrations were observed in lung tissue, as compared to plasma. Similarly, moxifloxacin distribution from plasma to lung lesion (k_25_=0.633 [79.8% RSE]) was faster than back to plasma (k_52_=0.318 [73.3% RSE]). Rate constant describing drug transfer across compartments reveal that moxifloxacin is less distributed in lung lesions as compared to healthy lung tissue.

|  |
| --- |
| Supplement Figure 4. Visual predictive check for moxifloxacin. The solid line represents the median, whereas the dashed lines represent the 5^th^ and 95^th^ percentiles of the observed (black dots) data. Dark and light grey shaded areas represent the 95% prediction intervals of the median, 5^th^ and 95^th^ percentiles of the predicted concentrations (N_simulations_=2000). |

Supplement Table 1. Summary of pharmacokinetic parameter estimates.

| Parameter | Fixed effects | Random effects | Fixed effects | Random effects | Fixed effects | Random effects | Fixed effects | | Random effects |
| --- | --- | --- | --- | --- | --- | --- | --- | --- | --- |
|  | (%RSE) | (%RSE) | (%RSE) | (%RSE) | (%RSE) | (%RSE) | (%RSE) | | (%RSE) |
|  | Isoniazid | | Rifampicin | | Pyrazinamide | | | Moxifloxacin | |
| k_a_ (h^-1^) | 0.209 (34.4) | 0.535 (92.2) | 0.236 (*fixed*) | - | 0.576 (66.9) | 0.756 (104) | 0.480 (43.8) | | 1.39 (105) |
| CL (l/h) | 52.4 (14.2) | 0.0216 (57.0) | 8.43 (12.2) | - | 2.6 (12.7) | 0.0503 (74.3) | 11.8 (20.5) | | 0.248 (77.7) |
| V_C_ (l) | 18.0 (0.419) | - | 44.6 (*fixed*) | 0.568 (0.00) | 34.5 (13.8) | 0.0431 (106) | 114 (*fixed*) | | - |
| Q (l/h) | 2.78 (3.92) | - | - | - | - | - | 2.9 (*fixed*) | | - |
| V_P_ (l) | 16.0 (0.800) | 0.112 (157) | - | - | - | - | 41.6 (*fixed*) | | - |
| F (%) | 1 (*fix*) | 0.172 (72.5) | - | - | - | - | - | | - |
| LAG_TIME_ (l/h) | - | - | 0.809 (42.5) | - | - | - | - | | - |
|  |  |  |  |  |  |  |  | |  |
| R_tissue/plasma_ | 0.907 (23.5) | - | 0.752 (15.0) | - | 0.628 (6.2) | - | - | | - |
| R_lesion/plasma_ | 0. 702 (21.7) | - | 0.478 (13.0) | - | 0.632 (7.56) | - | - | | - |
| k_plasma-tissue_ | - | - | - | - | - | - | 1.584 (37.3) | | - |
| k_tissue -plasma_ | - | - | - | - | - | - | 0.314 (50.1) | | - |
| k_plasma-lesion_ | - | - | - | - | - | - | 0.633 (79.8) | | - |
| k_lesion -plasma_ | - | - | - | - | - | - | 0.318 (73.3) | | - |
|  |  |  |  |  |  |  |  | |  |
| RUV_plasma_ | 1.19  (60.4) | - | 0.947 (48.9) | - | 0.0525 (60.5) | - | 0.11 (61.8) | | - |
| RUV­_tissue_ | 0.909 (25.1) | - | 0.344 (46.0) | - | 0.046 (27.0) | - | 0.331 (30.5) | | - |
| RUV_lesion_ | 0.852 (22.4) | - | 0.616 (13.0) | - | 0.149 (25.6) | - | 0.582 (25.3) | | - |
| Model structures | 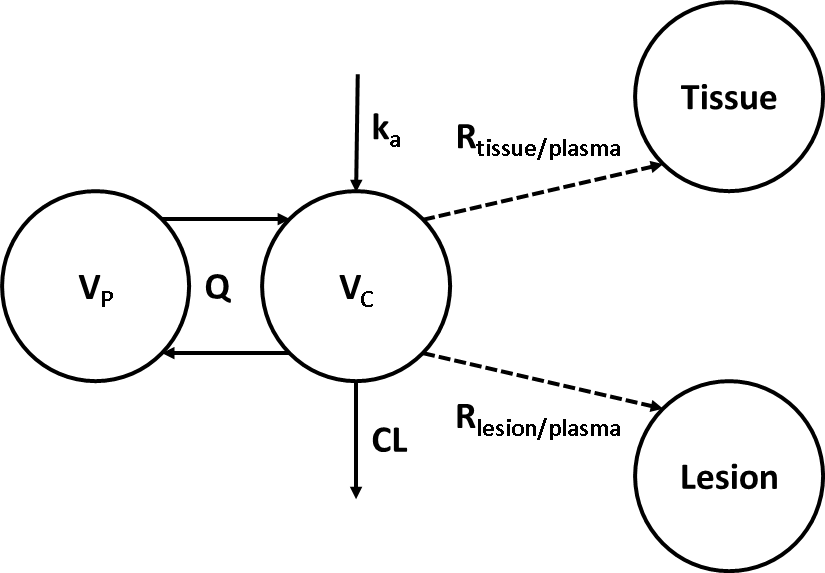 | | 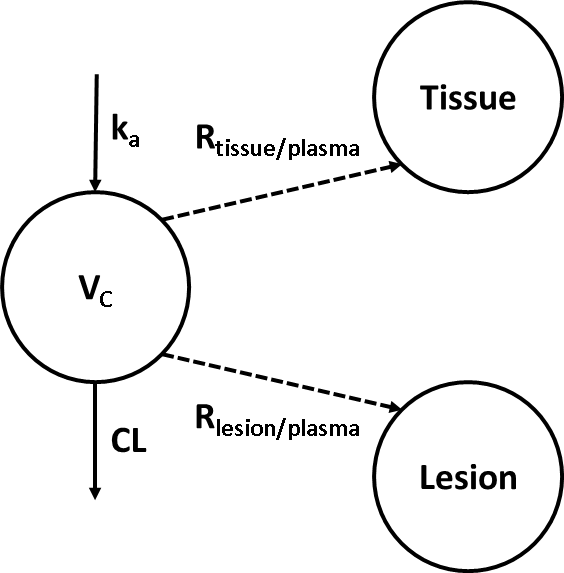 | | 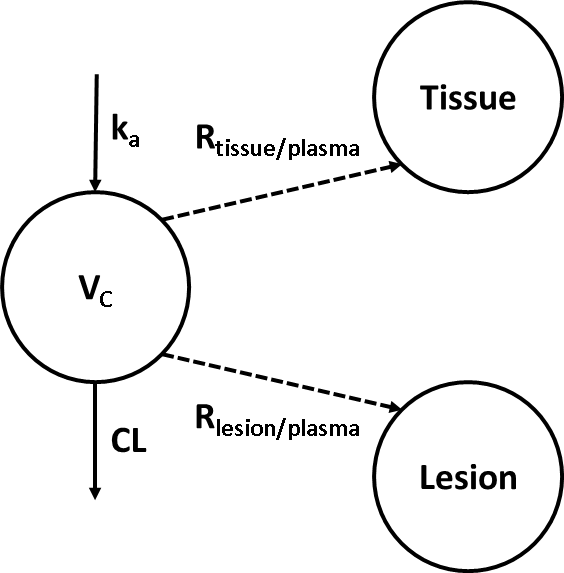 | | 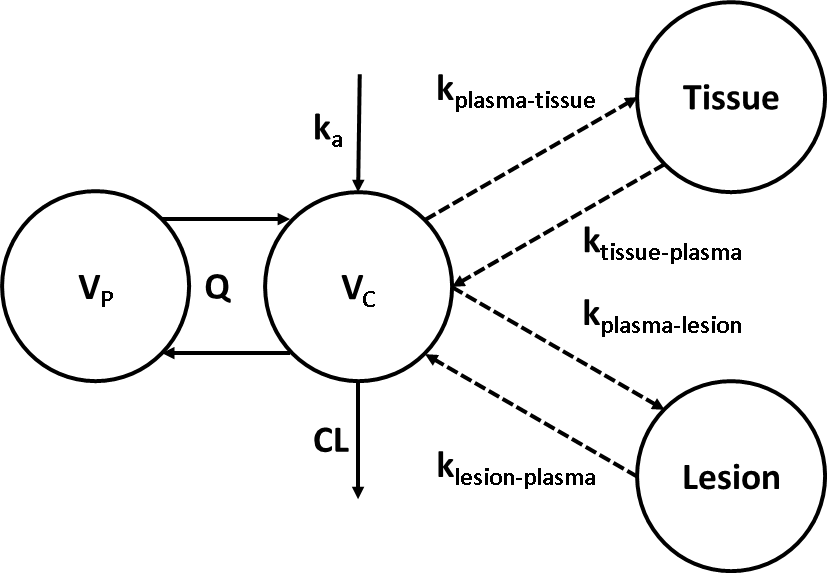 | | |

k_a_: absorption rate constant, CL: elimination clearance, V_C_: apparent volume of distribution central compartment, Q: inter-compartmental clearance, V_P_: apparent volume of distribution peripheral compartment, F: relative oral bioavailability, LAG_TIME_: lag-time on absorption rate constant, R_tissue/plasma_: tissue homogenate concentration over plasma concentration ration, R_lesion/plasma_: lesion homogenate concentration over plasma concentration ration, k_plasma-tissue_: rate constant from plasma to tissue homogenate compartment, k_tissue-plasma_: rate constant from tissue homogenate to plasma compartment, k_plasma-lesion_: rate constant from plasma to lesion homogenate compartment, k_lesion-plasma_: rate constant from lesion homogenate to plasma compartment. RUV: Residual variability (for plasma, tissue homogenate and lesion homogenate concentration) was described by an additive model using log-transformed data. Clearance and volume of distribution were allometrically scaled by body weight centred at 70 kg, with exponents of 0.75 and 1 for clearance and volume of distribution, respectively. RSE: Relative Standard Error, calculated as $100x\frac{standard deviation}{mean}$, where standard deviation and mean were derived from 1,000 non-parametric bootstrap runs.

## Population pharmacokinetic modelling discussion

*In-vivo* isoniazid, rifampicin and pyrazinamide lung exposure, tends to be lower when compared to in plasma (Figure 2). A study in rabbits reported similar decreased isoniazid, rifampicin and pyrazinamide exposures in lung tissue and lesion homogenate when compared to plasma^11^. Rifampicin exposure in epithelial lining fluid was also lower compared to in plasma 4 hours after administration to volunteers^12^. Isoniazid, and pyrazinamide levels tend to be higher in Epithelial Lining Fluid compared to in plasma, 4 hours after administration to volunteers on the other hand, although matrices differed substantially from each other^13,14^.

*In-vivo* moxifloxacin lung exposure, tends to be lower when compared to in plasma (Figure 2) which was in line a previous study in rabbits and in patient using *ex-viv*o microdialysis after surgical resection^11,15^. However, interpretation of moxifloxacin lung tissue and lesion homogenate data requires caution as the drug does not distribute well through both cellular regions and the acellular caseum^3,16^. An *in-vitro* hollow fiber experiment with moxifloxacin exposures mimicking lung lesion homogenate exposures might therefore over predict the anti-tuberculosis activity for bacteria residing in the cellular regions and acellular caseum. This may also explain the shorter time to culture negativity, yet higher treatment failure rates, when moxifloxacin was replacing isoniazid or ethambutol in the standard regimen in a clinical setting ^17^. *In-vivo* mimicking drug profiles in the hollow fibre experiments of infection were therefore limited to standard therapy drugs isoniazid, rifampicin and pyrazinamide.

Due to the limited sample size and scope of the current investigation, PK models should be treated primarily as descriptive and are not intended for other applications. Moreover, one of the limitations of our analysis was the lack of information on the covariates for the implementation of the isoniazid prior model. Details on the acetylator status of each subject was not available. Therefore, the prior for elimination clearance was uninformative which provided adequate fitting of the PK plasma profile and consequently of drug levels in lung tissue and lesion.

# References

1 Gao, S. *et al.* Rapid and sensitive method for simultaneous determination of first-line anti-tuberculosis drugs in human plasma by HPLC-MS/MS: Application to therapeutic drug monitoring. *Tuberculosis (Edinb)* **109**, 28-34, doi:10.1016/j.tube.2017.11.012 (2018).

2 Govender, K., Adamson, J. H. & Owira, P. The development and validation of a LC-MS/MS method for the quantitation of metformin, rifampicin and isoniazid in rat plasma using HILIC chromatography. *J Chromatogr B Analyt Technol Biomed Life Sci* **1095**, 127-137, doi:10.1016/j.jchromb.2018.07.041 (2018).

3 Prideaux, B. *et al.* The association between sterilizing activity and drug distribution into tuberculosis lesions. *Nature medicine* **21**, 1223-1227, doi:10.1038/nm.3937 (2015).

4 Clewe, O., Goutelle, S., Conte, J. E., Jr. & Simonsson, U. S. A pharmacometric pulmonary model predicting the extent and rate of distribution from plasma to epithelial lining fluid and alveolar cells--using rifampicin as an example. *European journal of clinical pharmacology* **71**, 313-319, doi:10.1007/s00228-014-1798-3 (2015).

5 Goutelle, S. *et al.* Population modeling and Monte Carlo simulation study of the pharmacokinetics and antituberculosis pharmacodynamics of rifampin in lungs. *Antimicrobial agents and chemotherapy* **53**, 2974-2981, doi:10.1128/AAC.01520-08 (2009).

6 Seng, K. Y. *et al.* Population pharmacokinetic analysis of isoniazid, acetylisoniazid, and isonicotinic acid in healthy volunteers. *Antimicrobial agents and chemotherapy* **59**, 6791-6799, doi:10.1128/AAC.01244-15 (2015).

7 Schipani, A. *et al.* A simultaneous population pharmacokinetic analysis of rifampicin in Malawian adults and children. *Br J Clin Pharmacol* **81**, 679-687, doi:10.1111/bcp.12848 (2016).

8 Smythe, W. *et al.* A semimechanistic pharmacokinetic-enzyme turnover model for rifampin autoinduction in adult tuberculosis patients. *Antimicrobial agents and chemotherapy* **56**, 2091-2098, doi:10.1128/AAC.05792-11 (2012).

9 Wilkins, J. J. *et al.* Variability in the population pharmacokinetics of pyrazinamide in South African tuberculosis patients. *European journal of clinical pharmacology* **62**, 727-735, doi:10.1007/s00228-006-0141-z (2006).

10 Zvada, S. P. *et al.* Moxifloxacin population pharmacokinetics in patients with pulmonary tuberculosis and the effect of intermittent high-dose rifapentine. *Antimicrobial agents and chemotherapy* **56**, 4471-4473, doi:10.1128/AAC.00404-12 (2012).

11 Kjellsson, M. C. *et al.* Pharmacokinetic evaluation of the penetration of antituberculosis agents in rabbit pulmonary lesions. *Antimicrobial agents and chemotherapy* **56**, 446-457, doi:10.1128/AAC.05208-11 (2012).

12 Conte, J. E., Golden, J. A., Kipps, J. E., Lin, E. T. & Zurlinden, E. Effect of sex and AIDS status on the plasma and intrapulmonary pharmacokinetics of rifampicin. *Clin Pharmacokinet* **43**, 395-404 (2004).

13 Conte, J. E., Jr., Golden, J. A., Duncan, S., McKenna, E. & Zurlinden, E. Intrapulmonary concentrations of pyrazinamide. *Antimicrobial agents and chemotherapy* **43**, 1329-1333 (1999).

14 Conte, J. E., Jr. *et al.* Effects of gender, AIDS, and acetylator status on intrapulmonary concentrations of isoniazid. *Antimicrobial agents and chemotherapy* **46**, 2358-2364 (2002).

15 Heinrichs, M. T. *et al.* Moxifloxacin target site concentrations in patients with pulmonary TB utilizing microdialysis: a clinical pharmacokinetic study. *The Journal of antimicrobial chemotherapy* **73**, 477-483, doi:10.1093/jac/dkx421 (2018).

16 Sarathy, J. P. *et al.* Prediction of Drug Penetration in Tuberculosis Lesions. *ACS Infect Dis* **2**, 552-563, doi:10.1021/acsinfecdis.6b00051 (2016).

17 Gillespie, S. H. *et al.* Four-month moxifloxacin-based regimens for drug-sensitive tuberculosis. *The New England journal of medicine* **371**, 1577-1587, doi:10.1056/NEJMoa1407426 (2014).
